# Nutrient Intake and Depression Symptoms in Spanish Children: The ANIVA Study

**DOI:** 10.3390/ijerph13030352

**Published:** 2016-03-22

**Authors:** Nuria Rubio-López, María Morales-Suárez-Varela, Yolanda Pico, Lorenzo Livianos-Aldana, Agustín Llopis-González

**Affiliations:** 1 Unit of Public Health, Hygiene and Environmental Health, Department of Preventive Medicine and Public Health, Food Science, Toxicology and Legal Medicine, University of Valencia, Valencia 46100, Spain; nuria.rubio@uv.es (N.R.-L.); maria.m.morales@uv.es (M.M.-S.-V.); 2 Biomedical Research Center Network on Epidemiology and Public Health (CIBERESP), Madrid 28029, Spain; yolanda.pico@uv.es (Y.P.); lorenzo.livianos@uv.es (L.L.-A.); 3 Center for Advanced Research in Public Health (CSISP-FISABIO), Valencia 46010, Spain; 4 Food and Environmental Safety Research Group, Faculty of Pharmacy, University of Valencia, Valencia 46100, Spain; 5 Research Center on Desertification (CIDE, UV-CSIC-GV), Carretera Moncada-Náquera, Moncada 46113, Spain; 6 Department of Psychiatry and Clinical Psychology, La Fe University and Polytechnic Hospital, Valencia 46026, Spain; 7 Department of Psychiatry, University of Valencia, Valencia 46100, Spain

**Keywords:** nutrients intake, nutritional intake, nutrition, depressive symptoms, carbohydrates, children

## Abstract

The aim of this study was to examine the relationship between nutritional intake and depressive symptoms in Valencian schoolchildren. The ANIVA (*Antropometria y Nutricion Infantil de Valencia*) study is a descriptive cross-sectional study. During academic year 2013–2014, 710 schoolchildren aged 6–9 years were selected from eleven primary schools in Valencia (Spain). Children’s dietary intake was measured on three-day food records, completed by parents/guardians; children completed the 20-item Center for Epidemiologic Studies Depression Scale for Children (CES-DC) Questionnaire to measure depressive symptoms. Weight, height, and body mass index (BMI), and z-scores were evaluated in all subjects. Nutrient adequacy was assessed using Spanish dietary recommended intakes (DRIs); 20.70% of the sample presented depressive symptoms. We identified a positive association between children with depressive symptoms and non-depressive symptoms for thiamin, vitamin K, and bromine (*p* < 0.05), and a negative association for protein, carbohydrates, pantothenic acid, biotin, vitamin B_12_ and E, zinc, manganese, cobalt, and aluminum (*p* < 0.05). Statistically significant differences were found between both groups according to the DRIs for intakes of total energy (*p* = 0.026), fiber (*p* < 0.001), vitamin C (*p* < 0.001), vitamin E (*p* = 0.004), magnesium (*p* = 0.018), and iron (*p* = 0.013). Our results demonstrated that carbohydrates were the most closely associated factor with depressive symptoms, and highlight the potential significant public health implications of inadequate nutritional intake on schoolchildren’s mental health.

## 1. Introduction

Childhood is a crucial period of physical, psychological, and social development [1]. Unfortunately, this period frequently coincides with onset of psychiatric illness [2]. Current global epidemiological data estimate that one child in five is expected to develop some form of mental health problem before reaching adulthood, and that 50% of all adult mental health problems develop in childhood and adolescence [3]. This highlights the importance of early prevention and intervention [4].

Adequate nutrition is a well-known relevant factor for children’s growth and development, not only in physiological terms, but also for optimal brain and cognitive function development [5,6]. However, evidence shows that young people’s diet quality has deteriorated significantly in recent decades [7,8,9,10,11]. Inadequate intake of energy or nutrients could have a detrimental effect on children’s health and predispose to childhood obesity, dental caries, poor academic performance, and lower self-esteem [12,13,14,15]. This decline in diet quality and an apparent parallel increase in the prevalence of adolescent depression have led to more interest being shown in the possible role of nutrition in the development or progression of depressive symptoms [7,16,17,18]. The above-cited articles have observed that eating a healthy diet is significantly associated with better emotional health (*p* < 0.001), while eating an unhealthy diet is significantly associated with greater emotional distress (*p* < 0.001).

An Australian [1] study showed that adolescents on a healthy diet were less likely to report symptomatic depression, while those who ate more processed ‘junk’ foods were more likely to report depression. Jacka *et al.* [19] reported that diet quality was negatively associated with adolescent mental health over time. The same study also reported that changes in diet quality were associated with changes in mental health, and improvements in diet quality were related with higher mental health scores upon follow-up, but not *vice versa*. A newly published Norwegian children study [20] found a significant relationship between eating patterns and mental health problems in young adolescents, independently of physical activity, sedentary activity, and several other background factors. This article showed that poorer diet quality was associated with declining psychological functioning. A Chinese [21] study reported a relationship between an unhealthy dietary pattern and emotional symptoms in 11–16-year-olds.

Very few studies have related nutritional intake to depressive symptoms in children in Spain, so more studies are needed to build knowledge in this field. Given the significance of both nutrition and mental health as public health concerns, and the paucity of research that examines associations between adequate dietary intake and mental health problems in Spanish children, the current study aimed to contribute to this emerging field by examining the relationship between nutritional intake and depressive symptoms in Valencian schoolchildren (6–9 years).

## 2. Materials and Methods 

### 2.1. Participants

ANIVA (*Antropometria y Nutrición Infantil de Valencia*; the Valencian Anthropometry and Child Nutrition) [22], a descriptive cross-sectional study, was conducted in schoolchildren aged 6–9 years who went to one of the eleven participating primary schools (see supplementary materials). According to a simple size calculation based on our preliminary data (Type I error: 0.05, power: 0.8), the estimated number of subjects was over 700. Children were selected by random cluster sampling in schools, and stratified by sex and type of school (*i.e.*, public *vs.* private). The latter factor was used as an approximate indicator of socio-economic status. Sampling was done in two stages: schools were selected from lists provided by the regional educational authorities. Then, classrooms and pupils were selected.

Data collection took place during academic year 2013–2014. The study was orally presented to the board of governors (*Consejo Escolar*) of each participating school. Next a letter was sent to the parents or guardians of all the children invited to participate, which outlined the study goals and procedures, and secured their written authorization. The inclusion criteria were: (a) children aged 6–9 years; (b) children who studied primary education at one of the eleven selected schools; and (c) parents or legal guardians had to agree about their child participating and give written informed consent. The exclusion criteria were: (a) clinical diagnosis of chronic disease with dietary prescription; (b) absence from school on the days arranged to take body weight and height measures; and (c) not properly completing the nutritional record. 

The initial sample included 873 children of both genders, of whom 12.8% did not want to participate (*N* = 112). The subjects who provided incomplete information, did not properly complete registration (*N* = 37), or did not present data on anthropometric measurements (*N* = 14) were removed. The participation rate was 81.3% and the resulting final sample comprised 710 children. 

All parents or legal guardians’s schoolchildren gave their informed consent for inclusion before they participated in the study. The study protocol complied with Declaration of Helsinki Guidelines and was approved by the *Secretaría Autonómica de Educación*, *Conserjería de Educación, Culturay Deporte* of the Generalitat Valenciana, Valencia, Spain (Ethics Committee 2014/29630). 

### 2.2. Examination Protocol and Measurements

Parents or guardians were interviewed during a questionnaire to acquire information on the child’s age, sex, medical history, medication, use of vitamin and mineral supplements, and other demographic characteristics. At the same time, they were provided with details of how to assess the food and drinks that their child consumed. They were asked to record estimated portion sizes for each ingested item. The same training was provided to the caregivers responsible for children in school dining halls. A visual guide was provided to improve the accuracy of portion size estimates, which was essential to obtain reliable data. Parents were asked to submit food labels with ingredients, brands, added ingredients and recipes for homemade dishes, whenever possible. They were given a telephone number for information and support, which they could call to help settle any issues that arose while completing the food records. 

### 2.3. Dietary Assessment

To carry out the dietary survey, parents and guardians were asked to record all the foods and drinks consumed by their child over a three-day period, including one non-working day (e.g., Sunday or Saturday) [23,24,25]. To calculate intakes of calories and macro- and micronutrients of known public health relevance, the researchers inputted data from the food records into an open-source computer software. This program (DIAL^®^, v2.16, Madrid, Spain) [26], developed by the Department of Nutrition and Dietetics at the Madrid Complutense University (Spain), has been previously validated in Spain to assess diets and to manage nutritional data. This open software includes a list of some of the enriched/fortified foods commonly available in Spain, to which other items can be added and foods can be added to the database. In this way, we were able to include the nutritional composition of packaged foods taken from food labels. 

### 2.4. Estimate of Nutrient Adequacy/Deficiency

Dietary Reference Intakes (DRIs) [27,28,29] include values for Recommended Dietary Allowances (RDAs), Estimated Average Requirements (EARs), Adequate Intakes (AIs), and Tolerable Upper Intake Levels (ULs), as well as Estimated Energy Requirements (EERs) for energy, and Acceptable Macronutrient Distribution Ranges (AMDRs) for macronutrients.

For each nutrient, children were categorized as being at risk of inadequate intake based on whether, or not, they met the corresponding nutritional targets [30] and DRIs [31] proposed for the Spanish population. Comparisons were made with the DRIs used in the USA to explore possible differences. The probability of adequate and usual intake of a given nutrient was calculated as follows: z-score = (estimated nutrient intake − EAR)/SD of EAR [32]. 

We used EARs for micronutrients, whenever available, and we took the AI values for the nutrients for which EARs were not determined. The percentage of energy provided by proteins, lipids, and carbohydrates were also calculated and compared with AMDRs. Using the data collected on consumed food, we made nutritional assessments for the following intakes: total energy (calories), carbohydrates, lipids, proteins, fiber, thiamin, riboflamin, niacin, pantothenic acid, vitamin B6, biotin, vitamin B12, C, D, and E; and minerals: calcium, phosphorus, magnesium, iron, zinc, iodine, selenium, and fluoride. For any nutrients presumed harmful (e.g., cholesterol), the opposite interpretation was applied. 

### 2.5. Anthropometric Measurements

During school hours, children’s height and weight were recorded with children standing barefoot in light clothing by the same person following standard procedures described by the World Health Organization (WHO) [33]. All the anthropometric measurements were obtained in duplicate and averaged. Weight was measured to the nearest 0.05 kg using a calibrated electronic load cell digital scale (OMRON BF511^®^, Tokyo, Japan) and height (in cm) was measured with a stadiometer (Seca 213^®^, Hamburg, Germany). Following the GPC Recommendations of the Spanish Ministry of Health and Social Policy, we took BMI as an index to calibrate nutritional status because it is an easy measure to obtain, is efficient and has been adopted internationally as a reasonable indicator of subcutaneous fat accumulation [34]. With these data, we calculated BMI-for-age (z-score) with the WHO Anthro software, v.3.2 (Geneva, Switzerland) [35]. Based on the obtained percentile ranking, BMI was used to classify children into one of four categories [36]: underweight (≤5th percentile), normoweight (>5th to <85th percentiles), overweight (≥85th to <95th percentiles), or obese (≥95th percentile). 

The tricipital skin-fold was measured at the top of the upper non dominant limb at a mid-point between the acromion and the olecranon, which was relaxed and placed in parallel with the axis (the technique of Durnin *et al.* [37]). Determinations were made in triplicate with a skinfold caliper (Holtein LTD, Pembs, UK) before calculating the mean.

### 2.6. Mental Health Measures

Depressive symptoms were evaluated using the Center for Epidemiological Studies Depression Scale for Children (CES-DC) Questionnaire [38], a 20-item self-report depression inventory with scores ranging from 0 to 60. Higher scores indicate increased depressive symptoms. Each response to an item was scored on a four-point Likert scale from 0 to 3 and participants were classified as depressed if they had a CES-DC score of ≥15 [38]. The internal reliability of the CES-DC in this study was high (Cronbach’s alpha = 0.86). No child was taking antidepressant medications. 

### 2.7. Statistical Analysis

Continuous variables are expressed as the means (standard deviations, SD), whereas categorical variables are expressed as frequency (percentages, %). The Kolmogorov-Smirnov test was used to determine the normality of the distribution of the examined variables. For the comparison of the means between groups, a one-way analysis of variance was used with the Bonferroni rule to correct for inflation in the type 1 error due to multiple *post hoc* comparisons. The Chi-square test was used to explore the association between categorical variables, and the two-sample Z-test for proportions for multiple *post hoc* comparisons. We made a multivariate comparison of the depressive symptoms in schoolchildren by a cluster analysis comparison of the demographic characteristics (residence, gender, family level of education, weight and height) and nutrient intakes (thiamin, manganese, pantothenic acid, zinc, biotin, cobalt, protein, carbohydrate, bromine, aluminum, vitamin B12, E, and K), which were significant (*p* < 0.05) in previous analyses when compared to children with and without depressive symptoms. A cluster dendrogram produces dendrograms (also called cluster trees) for hierarchical clustering. Dendrograms graphically present the information on which observations are grouped together at various levels of (dis)similarity. To the left of the dendrogram, each observation is considered its own cluster. Horizontal lines extend up for each observation, and at various (dis)similarity values, these lines are connected to the lines from other observations with a vertical line. Observations continue to combine until, to the right of the dendrogram, they are all grouped together. Long horizontal lines indicate a more distinct separation between groups. The long horizontal lines to the right of the dendrogram indicate that the groups represented by these lines are well separated from one another. Shorter lines indicate groups that are not as distinct. All the *p* values were two-tailed and statistical significance was set at the conventional cut-off of *p* < 0.05. Data were inputted into an Excel spreadsheet using a double-data entry to minimize risk of errors, and were then transferred to the IBM SPSS version 17.0 software (SPSS Inc., Chicago, IL, USA).

## 3. Results

Table 1 reports the demographic characteristics of the study sample, which included 710 schoolchildren. The sample comprised 372 girls (52.39%) and 338 boys (47.61%). In 20.70% (*n* = 147), students were classified as having depressive symptoms with a CES-DC score of ≥15. Mean age, mean tricipital fold, BMI, and nationality were similar in the depressive symptoms and non-depressive symptoms groups. However, when gender, height, weight, BMI-for-age, residence, and low family level of education were analyzed between both groups, a statistically significant difference (*p* < 0.05) was observed.

Macronutrients, micronutrients, and minerals intake were compared between the children with depressive and non-depressive symptoms, as shown in Table 2, together with the DRIs (EER, EAR, or AI) for children. Statistically significant differences were found between both groups for intake of protein, carbohydrates, pantothenic acid, biotin, vitamin B12,vitamin E, zinc, manganese, cobalt, aluminum, and bromine (*p* < 0.05), and was lower in the children with depressive symptoms. Statistically significant differences were also found for thiamin and vitamin K (*p* < 0.05), but intake was lower in the non-depressive group. More than 90% children in both groups reported inadequate intake of carbohydrates, fiber, and fluoride. More than 50% indicated inadequate vitamins D, vitamin E, zinc, and iodine intakes. We identified statistically significant differences between the children with depressive symptoms and non-depressive symptoms according to the DRIs for the intakes of total energy (*p* = 0.026), vitamin C (*p* < 0.001), vitamin E (*p* = 0.004), magnesium (*p* = 0.018), and iron (*p* = 0.013), which were lower in the children with depressive symptoms, while fiber (*p* < 0.001) was higher in the same children.

**Figure 1:**
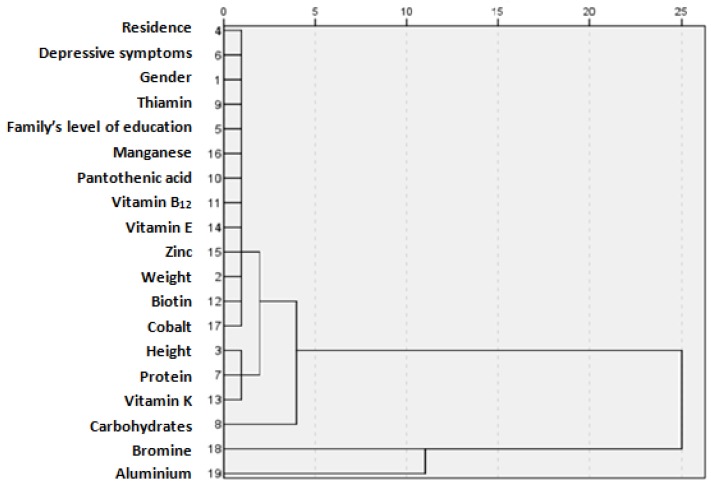
Hierarchical cluster: dendrogram using depressive symptoms in schoolchildren per demographic characteristic and nutrient intake.

As a result of the hierarchical cluster multivariate analysis, in which residence, gender, thiamin, family level of education, manganese, pantothenic acid, vitamin B12, vitamin E, zinc, weight, biotin, cobalt, height, protein, vitamin K, carbohydrates, bromine, and aluminum were valued, we obtained two clusters: the first was more associated with children’s depressive symptoms, along with their personal characteristics (gender, weight, height), environment (urban/rural residence, parents’ level of education), and nutrients stood out (thiamin, manganese, pantothenic acid, vitamin B_12_, vitamin E, zinc, biotin, cobalt, protein, vitamin K). Finally, carbohydrates were grouped with the previous nutrients. The second cluster was not directly related with children’s depressive symptoms, and bromine and aluminum intake were found to be less related with depressive symptoms compared with the other study variables (Figure 1).

## 4. Discussion

Childhood depression has a social and health impact on society, so its early detection is priority [39]. In this cross-sectional study of Spanish schoolchildren, we identified that 20.7% of the sample presented depressive symptoms. This is the first Spanish schoolchildren study to evaluate such prevalence in this age group (6–9 year-olds), which is a similar finding to that of Steinhausen *et al.* [40], who obtained a result of 23.6%. Other studies have indicated prevalence rates that ranged from 0.3% to 6.4% [41,42]. In our study, the children with depression symptoms came from families with lower levels of education (*p* < 0.001), rural residence (*p* = 0.037), and female gender (*p* = 0.005), compared to those without depression symptoms. Some authors have reported that depression in childhood is more frequent in girls than in boys [43,44], although this difference has not always been observed [41,45]. Most of the literature demonstrates high depression prevalence in overweight and obese children [46,47]. We found a similar overweight and obesity prevalence between both groups [48,49]. It remains unclear whether depression leads to obesity as a response to changing appetite, or if obesity contributes to depressive disturbances [50].

Our findings suggested that children aged 6–9 years showed scant compliance with the nutritional goals set by the DRIs for the Spanish population [30,31] (more than 25% showed inadequate intake for carbohydrates, fiber, folic acid, vitamin D, vitamin E, calcium, zinc, iodine, and fluoride, irrespective of the group they belonged to). It is worth stressing that intake estimates below recommendations did not indicate nutrient deficiencies as recommended intakes far exceeded the mean requirement. However, they are useful for indicating potential deficiencies in children with depressive symptoms, which will increase the larger the differences between those calculated according to real and recommended intakes become (DRIs). True deficiency statuses should be diagnosed by other means, especially biochemical analyses [51,52,53].

Several studies [54,55,56] have established that poor quality diet, which lacks nutrient-dense foods, may lead to nutrient deficiencies, which have been associated with mental health issues. Nutrition possibly plays a decisive role in onset of depression, and in its severity and duration. Numerous food patterns that precede depression have been identified in depression, such as wanting to eat lots of sweet foods, skipping meals, or having no appetite [57].

In the present study, children with depressive symptoms reported a lower intake of carbohydrates compared to the non-depressive symptoms group. Carbohydrates play an important role in structure and organism functioning, affect mood and behavior [57], and are the most important source of energy [58]. Carbohydrate intake affects the nervous system because they supply glucose and energy, and affect neurotransmitter synthesis in the brain and sympathetic nervous system activation. As a result, carbohydrate ingestion has a positive effect on several human behaviors, including appetite, sleep, activity, mood, cognition, and physical performance [59], which can even have an influence when depressive symptoms are absent. Eating meals rich in carbohydrates triggers the release of insulin. Insulin helps blood sugar enter cells, where it can be used for energy and to simultaneously trigger the entry of tryptophan to the brain. In the brain, tryptophan affects the levels of neurotransmitters [57] because it is the precursor of brain serotonin. Individuals with lower levels of brain serotonin are considered vulnerable to depression [56]. Unlike other studies [56,60] fiber intake in our study was lower in the children without depressive symptoms. 

B-group vitamins have been found to be implicated in the development of depression via the metabolism of neurotransmitters [61]. Our data indicated that schoolchildren with depressive symptoms mostly had lower intakes of vitamins than those without depressive symptoms; 42.16% of those with depressive symptoms displayed inadequate folic acid intake according to the DRIs [57]. Folic acid intake is very important because the active metabolite of folate is involved in the methylation of homocysteine, and also in methionine production, required for several important signal-transduction pathways that involve monoamine neurotransmitters. So deficiency in folate could raise homocysteine levels, which have been associated with depression [61,62]. Low folate and vitamin B12 status has been found in studies conducted with depressive patients, and an association between depression and low levels of these two vitamins has been found in studies conducted with the general population [63].

According to previous studies [56,64], antioxidants are important for preventing and treating depression because they help reduce the oxidative stress and cell damage caused by free radicals [65]. This occurs with vitamin C, which is thought to be effective in depression given its role in oxidative processes [50,66]. We found that vitamin C intake was lower in schoolchildren with depressive symptoms, and we identified a statistically significant difference according to the DRIs (*p* < 0.001). More than 50% of all our schoolchildren presented vitamin D and E intakes below the DRIs [56]. Ataie-Jafaei *et al.* [64] and Llewellyn *et al.* [67] reported significant associations between vitamin D deficiency and self-reported psychiatric distress, like depression. We found no significant associations between them, but identified a statistically significant difference only for vitamin E in both the depressive symptoms and non-depressive symptoms groups according to the DRIs (*p* = 0.004), although the intake of this vitamin was lower compared to the non-depressive symptoms group (*p* = 0.014). Low levels of vitamin E indicate fewer antioxidant defenses against lipid peroxidation, which increase in depression [68].

Apart from antioxidants, certain minerals (magnesium, calcium, zinc, manganese, and iron) are important for preventing and treating depression [56], and have been inversely associated with prevalence of depressive symptoms [69]. The intake of these nutrients was lower in the depressive symptoms group than the non-depressive symptoms one. Magnesium insufficiency leads to depression as a result of the neuron damage, which occurs when the magnesium requirements of neurons are not met [50]. Inadequate dietary zinc, iron, and manganese intake contributes to depressive symptoms [50,70]. They all contribute to brain function [71].

The intakes of calcium, iodine, and fluoride were well below those recommended in both groups, which coincided with other authors [72,73], but no differences were found between groups. 

Our findings highlighted the potential significant public health implications of inadequate nutritional intake on schoolchildren’s mental health. Thus, surveillance of dietary intake may enable early detection and the prevention of nutritional deficits, which are of vital importance for children’s proper growth and mental development.

### Study Limitations

This study is not without its study limitations, the first of which is our small sample size. The data we had available were insufficient to establish a clear relationship between nutritional adequacy and depressive symptoms. Future large-scale studies on this issue are necessary. Second, a cross-sectional study does not differentiate between cause and effect; we cannot exclude the possibility of depression also being influenced by some biological or functional pathway, nutritional habits, *etc.* Further research based on longitudinal studies is needed to examine the causal association between nutritional impairment and depressive symptoms. We believe that our study offers strong internal validity given the low attrition rate obtained. We are confident that the self-reported information employed for the nutrition assessment is of good quality. Parents and schools were very interested in the study, and were extensively trained and supported to complete food records.

## 5. Conclusions 

Nutritional inadequacy plays an important role in mental health and poor nutrition, and may contribute to the pathogenesis of depression. Our data identify that carbohydrate was the most closely associated factor with depressive symptoms in schoolchildren. Other nutrients, including dietary antioxidants and minerals, also have strong biological plausibility in affecting normal brain function and modulating mood. In demographic terms, schoolchildren were at high risk of depressive symptoms if their family’s level of education was lower and their residence was rural. It is also important to highlight the importance of designing nutrition education programs to enable the population in general, and schoolchildren in particular, to be made aware of, and to prepare for, healthy dietary habits. In this way, schoolchildren and parents will acquire excellent knowledge to help prevent disease and promote health.

## Figures and Tables

**Table 1:** Schoolchildren’s demographic characteristics based on the depressive symptoms classification.

Variables	Non Depressed Symptoms (*n* = 563) Mean ± SD or n (%)	95% CI	Depressed Symptoms (*n* = 147) Mean ± SD or n (%)	95% CI	*p* Value
Age (years)	8.21 ± 1.32	8.10–8.32	8.08 ± 1.14	7.90–8.26	0.274
Child’s gender					
Male	283 (50.27)	46.06–54.47	55 (37.41)	29.69–45.81	0.005
Female	280 (49.73)	45.53–53.94	92 (62.59)	54.19–70.31	0.005
Height (cm)	131.19 ± 8.74	130.47–131.91	127.89 ± 8.99	126.44–129.34	0.001
Weight (kg)	31.85 ± 7.71	31.21–32.49	29.60 ± 8.00	28.31–30.89	0.002
Tricipital fold (mm)	12.81 ± 4.54	12.43–13.19	12.60 ± 4.41	11.89–13.31	0.616
BMI for age (z-score)	1.19 ± 0.98	1.11–1.27	1.37 ± 0.97	1.21–1.53	0.047
BMI	18.31 ± 3.02	18.06–18.56	17.83 ± 3.20	17.31–18.35	0.091
Underweight	51 (9.06)	6.88–11.81	8 (5.44)	2.55–10.80	0.157
Normoweight	297 (52.75)	48.54–56.93	80 (54.42)	46.02–62.58	0.718
Overweight	114 (20.25)	17.05–23.86	25 (17.01)	11.51–24.27	0.377
Obesity	101 (17.94)	14.91–21.42	34 (23.13)	16.85–30.46	0.153
Residence					
Urban	311 (55.24)	51.02–59.38	67 (45.58)	37.41–53.98	0.037
Rural	252 (44.76)	40.61–48.98	80 (54.42)	46.02–62.58	0.037
Family’s level of education					
Low	93 (16.52)	13.60–19.91	42 (28.57)	21.58–36.70	0.001
Medium	277 (49.20)	45.00–53.41	55 (37.41)	29.69–45.81	0.118
High	193 (34.28)	30.39–38.39	50 (34.01)	26.54–42.34	0.951
Nationality					
Spanish	445 (79.04)	75.39–82.28	114 (77.55)	69.79–83.84	0.694
Other	118 (20.96)	17.72–24.61	33 (22.45)	16.16–30.21	0.694

**N**otes: BMI: Body Mass Index; SD: Standard Deviation; CI: Confidence Interval; *p* value < 0.05: was considered statistically significant.

**Table 2:** Nutrient intake and nutrient inadequacy in children with depressive and non-depressive symptoms.

Nutrients	Non Depressed Symptoms (*n* = 563) Mean ± SD	Depressed Symptoms (*n* = 147) Mean ± SD	*p* value
Total energy (kcal/day)	2155.47 ± 344.45	2110.75 ± 340.87	0.160
Percentage with intake <EER (1900)	29.9	39.2	0.026
Protein (g/day)	85.74 ± 14.87	82.63 ± 12.97	0.021
Percentage with intake < EAR (10%–15% TEV)	0.0	0.0	-
Carbohydrates (g/day)	218.43 ± 41.85	210.68 ± 41.73	0.045
Percentage with intake < EAR (50%–60% TEV)	94.2	96.4	0.238
Lipids (g/day)	95.48 ± 21.50	95.49 ± 18.37	0.996
Percentage with intake < EAR (30%–35% TEV)	2.0	2.3	0.238
Fiber (g/day)	13.78 ± 4.36	14.42 ± 3.97	0.107
Percentage with intake < IA (25 mg/day)	98.13	92.3	0.001
Thiamin (mg/day)	1.37 ± 0.35	1.43 ± 0.19	0.045
Percentage with intake < EAR (0.8 mg/day)	5.78	6.41	0.904
Riboflavin (mg/day)	1.82 ± 0.50	1.86 ± 0.49	0.386
Percentage with intake < EAR (1.2 mg/day)	7.17	9.82	0.324
Niacin (mg/day)	34.04 ± 7.36	33.28 ± 6.64	0.256
Percentage with intake < EAR (12 mg/day)	0.17	0.00	-
Pantothenic acid (mg/day)	5.46 ± 1.05	5.26 ± 1.12	0.042
Percentage with intake < AI (3 mg/day)	0.89	1.36	0.962
Vitamin B_6_ (mg/day)	1.96 ± 0.63	2.06 ± 0.50	0.072
Percentage with intake < EAR (1.4 mg/day)	15.07	10.20	0.129
Biotin (µg/day)	27.23 ± 0.86	26.45 ± 0.37	0.001
Percentage with intake < EAR (12)	1.60	3.6	0.286
Folic acid (µg/day)	236.03 ± 66.503	227.50 ± 68.37	0.169
Percentage with intake < EAR (200)	35.84	42.16	0.159
Vitamin B_12_ (µg/day)	5.89 ± 3.36	5.21 ± 1.38	0.016
Percentage with intake < EAR (1.5 µg/day)	0.0	1.36	-
Vitamin C (mg/day)	105.99 ± 38.51	99.14 ± 35.61	0.052
Percentage with intake < EAR (55)	6.10	18.72	0.001
Vitamin A (µg/day)	481.90 ± 110.36	461.71 ± 116.56	0.051
Percentage with intake < EAR (400 µg/day)	8.89	12.44	0.217
Vitamin D (µg/day)	2.71 ± 3.20	2.98 ± 2.24	0.336
Percentage with intake < EAR (5 µg/day)	83.81	80.25	0.304
Vitamin E (mg/day)	8.13 ± 3.55	7.34 ± 3.03	0.014
Percentage with intake < EAR (8 mg/day)	52.80	66.18	0.004
Vitamin K (µg/day)	114.61 ± 51.22	129.10 ± 51.79	0.002
Percentage with intake < EAR (55 µg/day)	0.0	0.0	-
Calcium (mg/day)	940.99 ± 235.88	934.50 ± 296.60	0.779
Percentage with intake < EAR (800 mg/day)	28.83	35.72	0.087
Phosphorus (mg/day)	1396.76 ± 251.51	1400.29 ± 333.56	0.888
Percentage with intake < EAR (700 mg/day)	0.53	1.36	0.607
Magnesium (mg/day)	287.36 ± 52.43	279.75 ± 74.97	0.156
Percentage with intake < IA (180 mg/day)	5.06	10.23	0.018
Iron (mg/day)	13.86 ± 4.19	13.28 ± 3.54	0.124
Percentage with intake < EAR (9 mg/day)	9.15	16.21	0.013
Zinc (mg/day)	9.60 ± 1.84	9.09 ± 1.76	0.003
Percentage with intake < EAR (10 mg/day)	72.41	77.38	0.213
Iodine (µg/day)	95.74 ± 28.52	91.07 ± 28.89	0.078
Percentage with intake < EAR (90 µg/day)	48.73	57.11	0.067
Fluoride (µg/day)	211.93 ± 60.00	205.11 ± 78.84	0.253
Percentage with intake < IA (1000 µg/day)	93.61	96.56	0.166
Selenium (µg/day)	107.53 ± 28.22	105.40 ± 26.43	0.409
Percentage with intake < EAR (30 µg/day)	0.0	0.0	-
Manganese (mg day)	3.09 ± 2.38	2.63 ± 1.26	0.024
Percentage with intake < IA (2 mg/day)	6.83	9.84	0.250
Cobalt (µg/day) *	20.22 ± 36.10	11.88 ± 21.09	0.007
Aluminum(µg/day) *	497.84 ± 208.57	453.03 ± 201.31	0.020
Bromine (µg/day) *	432.08 ± 338.94	498.34 ± 445.74	0.049

Notes: EER: Estimated Energy Requirements; EAR: Estimated Average Requirements; AI: Adequate Intakes; SD: Standard Deviation; CI: Confidence Interval; *p* value < 0.05: was considered statistically significant; * cobalt, aluminum, and bromine DRIs were not determined.

## Supplementary Materials

They are available online at www.mdpi.com/1660-4601/13/3/352/s1, ANIVA Study: The Food Intake Record Questionnaire.

## Abbreviations

The following abbreviations are used in this manuscript:

BMI	Body Mass Index
CES-D	Center for Epidemiologic Studies Depression Scale
DRI	Dietary Reference Intakes
ANIVA	Antropometria y Nutrición Infantil de Valencia
RDA	Recommended Dietary Allowances
EAR	Estimated Average Requirements
AI	Adequate Intakes
UL	Tolerable Upper Intake Levels
EER	Estimated Energy Requirements
AMDR	Acceptable Macronutrient Distribution Ranges
WHO	World Health Organization
SD	Standard Deviation

## References

[1] Jacka F.N., Kremer P.J., Leslie E.R., Berk M., Patton G.C., Toumbourou J.W., Williams J.W. (2010). Associations between diet quality and depressed mood in adolescents: Results from the Australian Healthy Neighbourhoods Study. Aust. N. Z. J. Psychiatry.

[2] Kessler R.C., Berglund P., Demler O., Jin R., Merikangas K.R., Walters E.E. (2005). Lifetime prevalence and age-of-onset distributions of DSM-IV disorders in the National Comorbidity Survey Replication. Arch. Gen. Psychiatry.

[3] Belfer M.L. (2008). Child and adolescent mental disorders: The magnitude of the problem across the globe. J. Child Psychol. Psychiatry.

[4] Hestetun I., Svendsen M.V., Oellingrath I.M. (2015). Associations between overweight, peer problems, and mental health in 12–13-year-old Norwegian children. Eur. Child Adolesc. Psychiatry.

[5] Bourre J.M. (2004). Roles of unsaturated fatty acids (especially omega-3 fatty acids) in the brain at various ages and during ageing. J. Nutr. Health Aging.

[6] Gomez-Pinilla F. (2008). Brain foods: The effects of nutrients on brain function. Nat. Rev. Neurosci..

[7] Kulkarni A.A., Swinburn B.A., Utter J. (2015). Associations between diet quality and mental health in socially disadvantaged New Zealand adolescents. Eur. J. Clin. Nutr..

[8] Adair L., Popkin B. (2005). Are child eating patterns being transformed globally?. Obes. Res..

[9] Nielsen S., Popkin B. (2003). Patterns and trends in food portion sizes, 1977–1998. JAMA.

[10] Nielsen S., Siega-Riz A., Popkin B. (2002). Trends in energy intake in U.S. between 1977 and 1996: Similar shifts seen across age groups. Obes. Res..

[11] Wang Z., Zhai F., Du S., Popkin B. (2008). Dynamic shifts in Chinese eating behaviors. Asia Pac. J. Clin. Nutr..

[12] Lobstein T., Baur L., Uauy R. (2004). Obesity in children and young people: A crisis in public health. Obes. Rev..

[13] Must A., Strauss R.S. (1999). Risks and consequences of childhood and adolescent obesity. Int. J. Obes. Relat. Metab. Disord..

[14] McCrindle B.W. (2015). Cardiovascular consequences of childhood obesity. Can. J. Cardiol..

[15] Maunder E.M., Nel J.H., Steyn N.P., Kruger H.S., Labadarios D. (2015). Added sugar, macro- and micronutrient intakes and anthropometry of children in a developing world context. PLoS ONE.

[16] Collishaw S., Maughan B., Goodman R., Pickles A. (2004). Time trends in adolescent mental health. J. Child Psychol. Psychiatry.

[17] Twenge J.M. (2011). Generational differences in mental health: Are children and adolescents suffering more, or less?. Am. J. Orthopsychiatry.

[18] West P., Sweeting H. (2003). Fifteen, female and stressed: Changing patterns of psychological distress over time. J. Child Psychol. Psychiatry.

[19] Jacka F.N., Kremer P.J., Berk M., de Silva-Sanigorski A.M., Moodie M., Leslie E.R., Pasco J.A., Swinburn B.A. (2011). A prospective study of diet quality and mental health in adolescents. PLoS ONE.

[20] Oellingrath I.M., Svendsen M.V., Hestetun I. (2014). Eating patterns and mental health problems in early adolescence–A cross-sectional study of 12–13-year-old Norwegian schoolchildren. Public Health Nutr..

[21] Weng T.T., Hao J.H., Qian Q.W., Cao H., Fu J.L., Sun Y., Huang L., Tao F.B. (2012). Is there any relationship between dietary patterns and depression and anxiety in Chinese adolescents?. Public Health Nutr..

[22] Morales-Suárez-Varela M., Rubio-López N., Ruso C., Llopis-Gonzalez A., Ruiz-Rojo E., Redondo M., Pico Y. (2015). Anthropometric status and nutritional intake in children (6–9 years) in Valencia (Spain): The ANIVA Study. Int. J. Environ. Res. Public Health.

[23] Barrett-Connor E. (1991). Nutrition epidemiology: How do we know what they ate?. Am. J. Clin. Nutr..

[24] Institute of Medicine (IOM) (2001). Dietary Reference Intakes: Applications in Dietary Assessment.

[25] Ortega R.M., Requejo A.M., López-Sobaler A.M., Ortega R.M., Requejo A.M. (2006). Modelos de cuestionarios para realización de estudios dietéticos en la valoración del estado nutricional. Nutriguía Manual de Nutrición Clínica en Atención Primaria.

[26] Ortega R.M., Lopez A.M., Andrés P., Requejo A.M., Aparicio A., Molinero L.M. (2008). DIAL Programa Para la Evaluación de Dietas y Gestión de Datos de Alimentación.

[27] Institute of Medicine (IOM). (2000). Dietary Reference Intakes: Applications in Dietary Assessment.

[28] Institute of Medicine (IOM) (2003). Dietary Reference Intakes: Applications in Dietary Planning.

[29] Murphy S.P., Barr S.I. (2011). Practice paper of the American Dietetic Association: Using the dietary reference intakes. J. Am. Diet. Assoc..

[30] Sociedad Española de Nutrición Comunitaria (SENC) (2001). Objetivos nutricionales para la población española. Span. J. Community Nutr..

[31] Federación Española de Sociedades de Nutrición, Alimentación y Dietética (FESNAD) (2010). Ingestas dietéticas de referencia (IDR) para la población española. Act. Diet..

[32] Carriquiry A.L. (1999). Assessing the prevalence of nutrient inadequacy. Public Health Nutr..

[33] (2006). World Health Organization Child Growth Standards: Length/Height-for-Age, Weight-for-Age, Weight-for-Length, Weight-for-Height and Body Mass Index for Age. http://www.who.int/childgrowth/standards/Technical_report.pdf.

[34] Onis M., Onyango A.W., Borghi E., Siyam A., Nishida C., Siekmann J. (2007). Development of a WHO growth reference for school-aged children and adolescents. Bull. World Health Organ..

[35] World Health Organization OMS Anthro, a Software for Assessing Growth and Development of the World’s Children (Version 3.2.2). http://www.who.int/childgrowth/software/es/.

[36] Centers for Disease Control and Prevention Defining Childhood Overweight and Obesity. http://www.cdc.gov/obesity/childhood/defining.html.

[37] Durnin J.V., Womersley J. (1974). Body fat assessed from total body density and its estimation from skinfold thickness: measurements on 481 men and women aged from 16 to 72 years. Br. J. Nutr..

[38] Faulstich M.E., Carey M.P., Ruggiero L., Enyart P., Gresham F. (1986). Assessment of depression in childhood and adolescence: An evaluation of the Center for Epidemiological Studies Depression Scale for Children (CES-DC). Am. J. Psychiatry.

[39] Najman J.M., Heron M.A., Hayatbakhsh M.R., Dingle K., Jamrozik K., Bor W., O’Callaghan M.J., Williams G.M. (2008). Screening in early childhood for risk of later mental health problems: A longitudinal study. J. Psychiatry Res..

[40] Steinhausen H.C., Winkler Metzke C. (2013). Prevalence of affective disorders in children and adolescents: Findings from the Zurich Epidemiological Studies. Acta Psychiatr. Scand. Suppl..

[41] Bernaras E., Jaureguizar J., Soroa M., Ibabe I., Cuevas C. (2013). Evaluation of the depressive symptomatology and the related variables in the school context. An. Psicol..

[42] Angold A., Erkanli A., Silberg J., Eaves L., Costello E.J. (2002). Depression scale scores in 8–17-year-olds: Effects of age and gender. J. Child Psychol. Psychiatry.

[43] Costello E.J., Mustillo S., Erkanli A., Keeler G., Angold A. (2003). Prevalence and development of psychiatric disorders in childhood and adolescence. Arch. Gen. Psychiatry.

[44] Stringaris A., Maughan B., Copeland W.S., Costello E., Angold A. (2013). Irritable mood as a symptom of depression in youth: Prevalence, developmental, and clinical correlates in the Great Smoky Mountains Study. J. Am. Acad. Child Adolesc. Psychiatry.

[45] Maughan B., Rowe R., Messer J., Goodman R., Meltzer H. (2004). Conduct disorder and oppositional defiant disorder in a national sample: Developmental epidemiology. J. Child Psychol. Psychiatry.

[46] Chung K.H., Chiou H.Y., Chen Y.H. (2015). Psychological and physiological correlates of childhood obesity in Taiwan. Sci. Rep..

[47] Sanders R.H., Han A., Baker J.S., Cobley S. (2015). Childhood obesity and its physical and psychological co-morbidities: A systematic review of Australian children and adolescents. Eur. J. Pediatr..

[48] Roohafza H., Kelishadi R., Sadeghi M., Hashemipour M., Pourmoghaddas A., Khani A. (2014). Are obese adolescents more depressed?. J. Educ. Health Promot..

[49] Morrison K.M., Shin S., Tarnopolsky M., Taylor V.H. (2014). Association of depression & health related quality of life with body composition in children and youth with obesity. J. Affect. Disord..

[50] Kaner G., Soylu M., Yüksel N., Inanç N., Ongan D., Başmısırlı E. (2015). Evaluation of nutritional status of patients with depression. Biomed Res. Int..

[51] Serra-Majem L., Ribas-Barba L., Pérez-Rodrigo C., Bartrina J.A. (2006). Nutrient adequacy in Spanish children and adolescents. Br. J. Nutr..

[52] Gibson R.S. (1990). Evaluation of Nutrient Intake Data Principles of Nutritional Assessment.

[53] Henríquez-Sánchez P., Díaz-Romero C., Rodríguez-Rodríguez E., López-Blanco F., Alvarez-Leon E., Díaz-Cremades J., Pastor-Ferrer M.C., Serra-Majem L. (2000). Evaluación bioquímica del estado nutricional de la población canaria (1997–1998). Arch. Latinoam. Nutr..

[54] Oddy W.H., Robinson M., Ambrosini G.L., O’Sullivan T.A., de Klerk N.H., Beilin L.J., Silburn S.R., Zubrick S.R., Stanley F.J. (2009). The association between dietary patterns and mental health in early adolescence. Prev. Med..

[55] O’Neil A., Quirk S.E., Housden S., Brennan S.L., Williams L.J., Pasco J.A., Berk M., Jacka F.N. (2014). Relationship between diet and mental health in children and adolescents: A systematic review. Am. J. Public Health.

[56] Khosravi M., Sotoudeh G., Majdzadeh R., Nejati S., Darabi S., Raisi F., Esmaillzadeh A., Sorayani M. (2015). Healthy and unhealthy dietary patterns are related to depression: A case-control study. Psychiatry Investig..

[57] Rao T.S., Asha M.R., Ramesh B.N., Rao K.S. (2008). Understanding nutrition, depression and mental illnesses. Indian J. Psychiatry.

[58] Jéquier E. (1994). Carbohydrates as a source of energy. Am. J. Clin. Nutr..

[59] Harvey-Anderson G. (1998). Carbohydrate, behavior and health. Bahrain Med. Bull..

[60] Fang C.Y., Egleston B.L., Gabriel K.P., Stevens V.J., Kwiterovich P.O., Snetselaar L.G., Longacre M.L., Dorgan J.F. (2013). Depressive symptoms and serum lipid levels in young adult women. J. Behav. Med..

[61] Blunden C.H., Inskip H.M., Robinson S.M., Cooper C., Godfrey K.M., Kendrick T.R. (2012). Postpartum depressive symptoms: The B-vitamin link. Ment. Health Fam. Med..

[62] Kim T.H., Choi J.Y., Lee H.H., Park Y. (2015). Associations between dietary pattern and depression in Korean adolescent girls. J. Pediatr. Adolesc. Gynecol..

[63] Coppen A., Bolander-Gouaille C. (2005). Treatment of depression: Time to consider folic acid and vitamin B12. J. Psychopharmacol..

[64] Ataie-Jafari A., Qorbani M., Heshmat R., Ardalan G., Motlagh M.E., Asayesh H., Arzaghi S.M., Tajadini M.H., Nejatinamini S., Poursafa P. (2015). The association of vitamin D deficiency with psychiatric distress and violence behaviors in Iranian adolescents: The CASPIAN-III study. J. Diabetes Metab. Disord..

[65] Frusciante L., Carli P., Ercolano M.R., Pernice R., Di Matteo A., Fogliano V., Pellegrini N. (2007). Antioxidant nutritional quality of tomato. Mol. Nutr. Food Res..

[66] Kohatsu W. (2005). Nutrition and depression. Explore.

[67] Llewellyn D.J., Langa K.M., Lang I.A. (2009). Serum 25-hydroxyvitamin D concentration and cognitive impairment. J. Geriatr. Psychiatry Neurol..

[68] German L., Kahana C., Rosenfeld V., Zabrowsky I., Wiezer Z., Fraser D., Shahar D.R. (2011). Depressive symptoms are associated with food insufficiency and nutritional deficiencies in poor community-dwelling elderly people. J. Nutr. Health Aging.

[69] Miki T., Kochi T., Eguchi M., Kuwahara K., Tsuruoka H., Kurotani K., Ito R., Akter S., Kashino I., Pham N.M. (2015). Dietary intake of minerals in relation to depressive symptoms in Japanese employees: The Furukawa Nutrition and Health Study. Nutrition.

[70] Lehto S.M., Ruusunen A., Tolmunen T., Voutilainen S., Tuomainen T.P., Kauhanen J. (2013). Dietary zinc intake and the risk of depression in middle-aged men: A 20-year prospective follow-up study. J. Affect. Disord..

[71] Bodnar L.M., Wisner K.L. (2005). Nutrition and depression: Implications for improving mental health among childbearing-aged women. Biol. Psychiatry.

[72] Manios Y., Moschonis G., Mavrogianni C., Bos R., Singh-Povel C. (2014). Micronutrient intakes among children and adults in Greece: The role of age, sex and socio-economic status. Nutrients.

[73] Durá-Travé T., Gallinas-Victoriano F. (2014). Dietary pattern among schoolchildren with normal nutritional status in Navarre, Spain. Nutrients.

